# Complete genome sequence of an *Escherichia coli* ST7366 strain harboring an extended-spectrum β-lactamase gene isolated from sewage water in Pakistan

**DOI:** 10.1128/mra.00793-24

**Published:** 2024-12-12

**Authors:** Jung Hun Lee, Saba Yasmin, Nam-Hoon Kim, Chanyeong Jeong, Byeonghyeon Kang, Gwangje Lee, Dae-Wi Kim, Rabaab Zahra, Sang Hee Lee

**Affiliations:** 1 National Leading Research Laboratory of Drug Resistance Proteomics, Department of Biological Sciences, Myongji University, Yongin, South Korea; 2 Department of Microbiology, Quaid-i-Azam University, Islamabad, Pakistan; 3 Department of Life Sciences, Jeonbuk National University, Jeonju, South Korea; Loyola University Chicago, Chicago, Illinois, USA

**Keywords:** *Escherichia coli*, extended-spectrum β-lactamase, plasmid, sewage

## Abstract

We announce the complete genome of *Escherichia coli* strain PEC1003 belonging to ST7366, isolated from sewage water in Pakistan. This strain harbored a plasmid carrying various antibiotic resistance genes, including extended-spectrum β-lactamase (ESBL), *bla*_CTX-M-15_, emphasizing the management and surveillance of ESBL-producing *E. coli* in the environment of developing countries.

## ANNOUNCEMENT

*Escherichia coli* is an opportunistic pathogen responsible for various infectious diseases (1). Pathogenic *E. coli* strains frequently acquire extended-spectrum β-lactamase genes via horizontal gene transfer (2). This phenomenon poses a significant health risk, particularly in developing countries (3).

*E. coli* PEC1003 was isolated from sewage water at Quaid-i-Azam University, Pakistan (33.7476°N, 73.1381°E). The sample was briefly centrifuged to remove particles, and 100 µL of the supernatant was spread onto a MacConkey agar plate containing cefotaxime (1 µg/mL). Pink-colored colonies were selected as *E. coli* candidates. *E. coli* PEC1003 was identified by biochemical analyses and *uidA* gene amplification. The isolate was cultured overnight at 37°C in Luria-Bertani medium containing ampicillin (100 µg/mL), and genomic DNA was extracted using the Qiagen MagAttract HMW DNA kit (Qiagen, Germany), following the manufacturer’s instructions. A hybrid sequencing approach employing MiSeq (Illumina, USA) and PacBio Sequel (Pacific Biosciences, USA) platforms was used to obtain the complete genome of the strain. MiSeq library was constructed using the TruSeq DNA Library LT kit (Illumina, USA). The library was sequenced on the Illumina MiSeq system with 2 × 300 bp paired-end reads using the MiSeq Reagent Kit v3. For the PacBio sequencing, the genomic DNA was sheared using Megaruptor3 (Diagenode, USA), and AMpureXP bead purification (Beckman Coulter, USA) was used to remove small fragments. The SMRTbell Express Template Preparation Kit (Pacific Biosciences, USA) was employed to construct the SMRTbell library. Sequel Sequencing Kit version 3.0 and SMRT Cell 1M version 2 were used to sequence the library, and the sequencing data were obtained using the sequel sequencing platform (Pacific Biosciences, USA). MiSeq sequencing data were quality controlled with Trimmomatic (version 0.36). Hybrid assembly was performed by Unicycler (version 0.4.9). Circulator (version 1.4.0) was employed to circularize contigs and rotate them as replication origin. The annotation of strain PEC1003 assembly was conducted using the Prokaryotic Genome Annotation Pipeline (version 6.8) (4). A total of 4,478 genes were predicted in the genome. Phylogroup and multi-locus sequence type of the stain were analyzed using the ClermonTyping and the pubMLST (Achtman scheme), respectively (5, 6). The Abricate (version 1.0.0) was employed to define its serotype, plasmid replicon sites, and the profiles of virulence factors (VFs) (https://github.com/tseemann/abricate). The typing of type-1 fimbrial adhesin (*fimH*) was carried out using the Fimtyper (7). The Resistance Gene Identifier was used to identify antibiotic resistance genes (ARGs) in the genome (8). Default parameters were used except where otherwise noted.

The genome of strain PEC1003 consists of two circular contigs: one chromosome (4,490,979 bp) and one FIB replicon plasmid pPEC1003 (137,057 bp). The sequencing depth of coverage was 745.7×. The strain was classified as ST7366 in phylogroup A, O184:H30 serotype, and FimH41 type. The plasmid harbors nine ARGs: *bla*_CTX-M-15_*, bla*_TEM-1_*, aph(3″)-Ib, aph (6)-Id, qnrS1, sul2, tet(A),* and two *dfrA14* genes. Table 1 indicates VF genes in the chromosome.

**TABLE 1:** Virulence factor genes in the chromosome of *E. coli* strain PEC1003

VF				
ECP^*a*^	Enterobactin	LEE-encoded T3SS^*b*^	Type 1 fimbriae	Etc^*c*^
*ykgK/ecpR, yagZ/ecpA, yagY/ecpB, yagX/ecpC, yagW/ecpD, agV/ecpE*	*entA, entB, entC, entD, entE, entF, entS, fepA, fepB, fepC, fepD, fepG, fes*	*espR4, espL1, espR1, espX1, espX5, espX4*	*fimH, fimG, fimF, fimD, fimC, fimI, fimA, fimE, fimB*	*csgB, csgF, csgG, aslA, csgD, fdeC, ompA*

^*a*^ ECP, *Escherichia coli* common pilus.

^*b*^ LEE- encoded T3SS, locus of enterocyte effacement encoded type III secretion system.

^*c*^ Etc, Agf, AslA, curli fibers/thin aggregative fimbriae, FedC, OmpA.

## Data Availability

The genome is available at the NCBI BioSample PRJNA1127693 within the BioProject SAMN42017831. The genome sequences are available in the GenBank accession number CP160643 and CP160644. The raw sequence data are available in the SRA SRX25452035 and SRX25456954.
